# Total Effective Xenoestrogen Burden in Serum Samples and Risk for Breast Cancer in a Population-Based Multicase–Control Study in Spain

**DOI:** 10.1289/EHP157

**Published:** 2016-05-20

**Authors:** Roberto Pastor-Barriuso, Mariana F. Fernández, Gemma Castaño-Vinyals, Denis Whelan, Beatriz Pérez-Gómez, Javier Llorca, Cristina M. Villanueva, Marcela Guevara, José-Manuel Molina-Molina, Francisco Artacho-Cordón, Laura Barriuso-Lapresa, Ignasi Tusquets, Trinidad Dierssen-Sotos, Nuria Aragonés, Nicolás Olea, Manolis Kogevinas, Marina Pollán

**Affiliations:** 1 Cancer and Environmental Epidemiology Unit, National Center for Epidemiology, Carlos III Institute of Health, Madrid, Spain; 2 Consortium for Biomedical Research in Epidemiology and Public Health (CIBERESP), Madrid, Spain; 3 Biosanitary Institute of Granada (ibs.GRANADA), University of Granada, Granada, Spain; 4 Center for Research in Environmental Epidemiology (CREAL), Barcelona, Spain; 5 Hospital del Mar Medical Research Institute (IMIM), Barcelona, Spain; 6 Pompeu Fabra University (UPF), Barcelona, Spain; 7 USA-Spain Fulbright Commission for Cultural, Educational and Scientific Exchange, Madrid, Spain; 8 Department of Biostatistics and Bioinformatics, Rollins School of Public Health, Emory University, Atlanta, Georgia, USA; 9 Division of Epidemiology and Computational Biology, School of Medicine, University of Cantabria, Santander, Spain; 10 Public Health Institute of Navarra, Pamplona, Spain; 11 Medicine Department, Autonomous University of Barcelona, Barcelona, Spain; 12 Medical Oncology Department, Hospital del Mar, Barcelona, Spain

## Abstract

**Background:**

Most studies on endocrine-disrupting chemicals and breast cancer have focused on single compounds and have produced inconclusive findings.

**Objectives:**

We assessed the combined estrogenic effects of mixtures of xenoestrogens in serum and their relationship to breast cancer risk.

**Methods:**

A total of 186 incident pretreatment breast cancer cases and 196 frequency-matched controls were randomly sampled from a large population-based multicase–control study in Spain. The total effective xenoestrogen burden attributable to organohalogenated xenoestrogens (TEXB-α) and endogenous hormones and more polar xenoestrogens (TEXB-β) was determined in serum samples using high-performance liquid chromatography and E-Screen bioassay. Odds ratios for breast cancer comparing tertiles of serum TEXB-α and TEXB-β were estimated using logistic models, and smooth risk trends were obtained using spline models.

**Results:**

Cases had higher geometric mean TEXB-α and TEXB-β levels (8.32 and 9.94 Eeq pM/mL, respectively) than controls (2.99 and 5.96 Eeq pM/mL, respectively). The fully adjusted odds ratios for breast cancer (95% confidence intervals) comparing the second and third tertiles of TEXB-α with the first tertile were 1.77 (0.76, 4.10) and 3.45 (1.50, 7.97), respectively, and those for TEXB-β were 2.35 (1.10, 5.03) and 4.01 (1.88, 8.56), respectively. A steady increase in risk was evident across all detected TEXB-α levels and a sigmoidal trend was observed for TEXB-β. Individual xenoestrogens showed weak and opposing associations with breast cancer risk.

**Conclusions:**

This is the first study to show a strong positive association between serum total xenoestrogen burden and breast cancer risk, highlighting the importance of evaluating xenoestrogen mixtures, rather than single compounds, when studying hormone-related cancers.

**Citation:**

Pastor-Barriuso R, Fernández MF, Castaño-Vinyals G, Whelan D, Pérez-Gómez B, Llorca J, Villanueva CM, Guevara M, Molina-Molina JM, Artacho-Cordón F, Barriuso-Lapresa L, Tusquets I, Dierssen-Sotos T, Aragonés N, Olea N, Kogevinas M, Pollán M. 2016. Total effective xenoestrogen burden in serum samples and risk for breast cancer in a population-based multicase–control study in Spain. Environ Health Perspect 124:1575–1582; http://dx.doi.org/10.1289/EHP157

## Introduction

Malignant breast tumors are the leading cause of cancer in women worldwide in terms of incidence and mortality (Ferlay et al. 2013). Despite efforts to elucidate breast cancer etiology, genetic determinants and well-established risk factors explain a limited amount of the global burden of this disease (Barnes et al. 2011; Howell et al. 2014; Sprague et al. 2008). It is noteworthy that most recognized determinants of breast cancer, such as reproductive history, alcohol intake, obesity, and use of hormone therapy, exert their effects, at least in part, by modifying the time and intensity of the exposure of the mammary gland to steroidal hormones (Brown and Hankinson 2015; Hilakivi-Clarke et al. 2013; MacMahon 2006; Renehan et al. 2015; Seitz et al. 2012).

Laboratory studies, specifically rodent models, support the implication of environmental pollutants in breast cancer development (Dhimolea et al. 2014; Rudel et al. 2007). Endocrine-disrupting chemicals (EDCs) are among the 17 chemical groups prioritized for evaluation in epidemiological studies of breast cancer (Rudel et al. 2014) because of their potential to act as xenoestrogens or to modulate estrogenic activity via different pathways [Gibson and Saunders 2014; World Health Organization/United Nations Environment Programme (WHO/UNEP) 2013]. Hundreds of EDCs are present in human breast tissue, but epidemiological evidence linking these substances with breast cancer is inconclusive (WHO/UNEP 2013). Most previous studies have focused on individual EDCs with weak estrogenic effects, thus failing to consider multiple exposures and interactions involving different EDCs and physiological hormones (Fernández et al. 2014). Functional tests measuring the combined estrogenic activity of mixtures of EDCs offer a promising approach for an aggregated exposure assessment. A case–control study reported a positive association between the combined effect of environmental estrogens in human adipose tissue and breast cancer risk (Ibarluzea et al. 2004). However, adipose tissue is difficult to obtain in population-based studies, and it would be of great practical value to assess the estrogenic potential of EDC mixtures present in blood samples (Rudel et al. 2014).

In the present study, we measured the combined estrogenic activity of mixtures of xenoestrogens in serum samples and evaluated its relationship to breast cancer risk in a subsample of cases and controls from a large population-based multicase–control study in Spain (MCC-Spain).

## Methods

### Study Population

MCC-Spain (http://www.mccspain.org) is a population-based multicase–control study conducted between 2008 and 2013 in 12 Spanish provinces to identify environmental, personal, and genetic factors related to five common cancers: breast, prostate, colorectal, stomach, and chronic lymphocytic leukemia. The study design has been previously reported (Castaño-Vinyals et al. 2015). Briefly, the study recruited 6,082 patients 20–85 years old with histologically confirmed incident cancer, including 1,750 breast cancers, 1,115 prostate cancers, 2,171 colorectal cancers, 492 gastroesophageal cancers, and 554 cases of leukemia, as well as a single set of 4,101 population controls. The response rates were 69% among breast cancer cases and 54% among female controls. All participants completed computer-assisted personal interviews on sociodemographic factors, self-reported anthropometric data, lifestyle, reproductive history, hormonal factors, medications, and personal and family medical history. Blood samples were collected from 76% of participants. The study was approved by the ethics committees of the participating institutions. Written informed consent was obtained from each participant.

For this analysis, we randomly selected 204 breast cancer cases from among those who agreed to donate blood samples in the provinces of Madrid, Barcelona, Navarra, and Cantabria; we also selected 204 female controls who were frequency-matched to cases by province, 5-year age interval, and 2-unit category of body mass index.

### Biochemical Analyses

We measured the total effective xenoestrogen burden (TEXB) in serum samples using a standardized bioassay for the combined estrogenic effect of mixtures of xenoestrogens (Fernández et al. 2004), which has been applied to extracts of adipose tissue, blood, and placenta (Fernandez et al. 2007; Ibarluzea et al. 2004; Lopez-Espinosa et al. 2009; Sonnenschein et al. 1995). Three milliliters of serum was added to the same volume of methanol, and the solution was extracted with 5 mL of hexane:ethyl ether (1:1 v/v). The organic phase was then passed through a Bond Elut PCB cartridge (Varian) that had previously been conditioned with 1.5 mL hexane. The obtained eluate was dried at reduced pressure under a stream of nitrogen. Dried serum extracts were reconstituted in 200 μL hexane, halved, and eluted in duplicate using high-performance liquid chromatography (HPLC). This semipreparative HPLC method was developed to efficiently separate organohalogenated lipophilic xenoestrogens [organochlorine pesticides and metabolites, polychlorinated biphenyls (PCBs), and halogenated bisphenols, among others] eluting in the α fraction from endogenous hormones and more polar xenoestrogens (nonhalogenated bisphenols, polyphenols, phytoestrogens, and mycoestrogens) eluting in the β fraction, using a normal-phase column and a gradient with two mobile phases [*n*-hexane (phase A) and *n*-hexane:methanol:2-isopropanol (40:45:15 v/v) (phase B)], with the most lipophilic compounds eluting in the shortest time. After HPLC fractionation, duplicated dry extracts of each fraction were combined, resuspended in experimental steroid-free medium (phenol red–free medium supplemented with 2.5 mL of charcoal-dextran fetal bovine serum), and tested for estrogenic activity with the E-Screen bioassay (Soto et al. 1995). The combined estrogenic activity of all compounds included in each fraction was analyzed from its proliferative effect on MCF-7 human breast cancer cells (kindly provided by A. Soto and C. Sonnenschein, Tufts University, Medford, MA). Each fraction extract was assayed at three different dilutions (1:1, 1:5, and 1:10), along with a negative control (experimental steroid-free medium) and a positive control (treated with 100 pmol of estradiol) in each culture plate. The proliferative effects of α and β fractions were calculated as the difference in MCF-7 cell proliferation between the fraction extract and the steroid-free control divided by the highest difference in proliferation between the estradiol-treated and steroid-free control cells. These relative proliferative effects were transformed into estradiol equivalent units by reading from a sigmoidal dose–response curve prepared with estradiol at concentrations of 0.1–1,000 pM, and they were expressed as the estradiol equivalent concentration in picomolar per milliliter of serum (Eeq pM/mL) that would produce the same cell proliferation in the bioassay (Fernandez et al. 2007). Thus, TEXB of the alpha fraction (TEXB-α) can be regarded as a biomarker of the combined estrogenic effect of mixtures of organohalogenated lipophilic xenoestrogens, whereas TEXB of the beta fraction (TEXB-β) represents the combined estrogenic activity of endogenous hormones and more polar xenoestrogens.

The limit of detection for TEXB-α and TEXB-β was 0.1 Eeq pM/mL, which corresponded to the minimum concentration needed to produce a significantly different proliferative effect from that observed in steroid-free control cells. For 6.3% and 3.7% of participants with TEXB-α and TEXB-β determinations below the limit of detection, respectively, a level equal to the limit of detection divided by the square root of 2 was imputed. TEXB-α and TEXB-β levels could not be determined in 44.2% and 35.1% of serum samples, respectively, because MCF-7 cells treated with their extracts grew less than steroid-free control cells, which hampered reading the proliferative effect in the estradiol dose-response curve. For quality control, 10 serum samples were analyzed in triplicate through independent extraction, HPLC fractionation, and E-Screen bioassay. The interassay coefficients of variation for TEXB-α and TEXB-β were 18.5% and 11.1%, respectively.

Specific organohalogenated compounds present in the HPLC α fraction, such as PCB-138, PCB-153, PCB-180, hexachlorobenzene (HCB), and *p*,*p*´-dichlorodiphenyldichloroethylene (*p*,*p*´-DDE), were quantified using high-resolution gas chromatography with microelectron capture detection and using *p*-chlorodibenzophenone as the internal standard. The limit of detection for all of these chemicals was set at 0.05 ng/mL, representing the smallest analyte amount that gave a signal-to-noise ratio > 3. For 5.2%, 1.8%, 3.4%, 8.4%, and 2.1% of participants with serum concentrations of PCB-138, PCB-153, PCB-180, HCB, and *p*,*p*´-DDE, respectively, below the limit of detection, a level equal to the limit of detection divided by the square root of 2 was imputed. Total cholesterol and triglycerides were enzymatically quantified in 10 μL of serum using a Cobas 400 analyzer (Roche), and total lipids were derived from the short formula based on these measured lipid species (Phillips et al. 1989).

### Statistical Analysis

Participants were grouped into tertiles of serum TEXB-α and TEXB-β levels based on their distributions among the controls. Odds ratios and 95% confidence intervals (CIs) for breast cancer comparing the second and third tertiles with the first tertile of serum TEXB-α and TEXB-β were estimated using logistic regression models. We also estimated the odds ratio for women with undetermined estrogenicity in the bioassay compared with all other women with determined estrogenicity. Tests for linear risk trends across serum TEXB-α and TEXB-β tertiles were performed by including an ordinal variable with the median level of each tertile among controls in logistic regression models. To further explore the shape of the dose–response relationships of serum TEXB-α and TEXB-β levels with breast cancer risk, we used restricted quadratic splines for log-transformed TEXB-α and TEXB-β levels with knots at the 10th, 50th, and 95th percentiles of their control distributions (the first knot was set at the 10th percentile to exceed levels below the limit of detection) (Greenland 1995). We also estimated odds ratios for breast cancer comparing tertiles of specific organohalogenated compounds (PCB-138, PCB-153, PCB-180, HCB, and *p*,*p*´-DDE) based on their control distributions.

Logistic regression models were fitted with increasing degrees of adjustment. The first model adjusted for province (Madrid, Barcelona, Navarra, or Cantabria), age (continuous), body mass index (continuous), education level (primary or less, high school, or college), and serum total lipid levels (continuous). The second model further adjusted for breast cancer risk factors, including smoking status (never, former, or current), number of births (nulliparous, 1–2, or ≥ 3), age at first birth (continuous), menopausal status (premenopausal or postmenopausal), use of hormone replacement therapy (never or ever), previous breast biopsy (no or yes), and family history of breast cancer (no, second-degree relative, or first-degree relative). Finally, the third model mutually adjusted serum TEXB-α and TEXB-β levels for each other. Effect modifications were contrasted by including interaction terms of serum TEXB-α and TEXB-β tertiles with each of the above covariates in logistic regression models. Analyses were performed using Stata v.13.1 (StataCorp) and R (v.2.15; R Project for Statistical Computing).

## Results

From the 408 randomly selected women (204 breast cancer cases and 204 controls), we excluded 5 prevalent or recurrent cases of breast cancer at baseline interview, 5 cases who initiated chemotherapy or hormone therapy before blood extraction, 1 case who withdrew initial consent, and 15 additional women (7 cases and 8 controls) with insufficient serum samples. Thus, the final sample included 186 incident pretreatment cases of breast cancer (166 invasive and 20 ductal carcinoma *in situ*) and 196 population-based controls with available serum samples for estrogenicity analyses. The mean age and mean body mass index among cases and frequency-matched controls were 59.8 years and 26.3 kg/m^2^, respectively. Compared with controls, cases were more likely to be nulliparous, ever smokers, and ever users of hormone therapy, and to have lower education levels and higher prevalences of breast biopsies and affected first-degree relatives; however, only the difference in the prevalence of breast biopsies was statistically significant (Table 1). The geometric mean serum levels of TEXB-α and TEXB-β were significantly higher in breast cancer cases (8.32 and 9.94 Eeq pM/mL, respectively) than in controls (2.99 and 5.96 Eeq pM/mL, respectively). Samples with undetermined estrogenicity in the bioassay were equally distributed among cases and controls. Regarding specific organohalogenated compounds, cases had marginally lower HCB concentrations and similar concentrations of PCB-138, PCB-153, PCB-180, and *p*,*p*´-DDE than controls (Table 1).

**Table 1:** Main characteristics and serum levels of total effective xenoestrogen burden and specific organohalogenated compounds in breast cancer cases and controls (*n* = 382).

Characteristic	Controls	Breast cancer cases	*p*-Value^*a*^
Number of women	196	186
Province
Madrid	84 (42.9)	71 (38.2)
Barcelona	34 (17.3)	33 (17.7)
Navarra	26 (13.3)	27 (14.5)
Cantabria	52 (26.5)	55 (29.6)
Age (years)	59.8 ± 10.7	59.7 ± 11.1
Body mass index^*b*^ (kg/m^2^)	26.2 ± 4.5	26.4 ± 4.5
Education level			0.40
Primary or less	94 (48.0)	102 (54.8)
High school	72 (36.7)	59 (31.7)
College	30 (15.3)	25 (13.5)
Smoking status^*b*^			0.28
Never	124 (63.3)	103 (55.6)
Former	33 (16.8)	41 (22.2)
Current	39 (19.9)	41 (22.2)
Number of births			0.38
Nulliparous	33 (16.9)	39 (21.1)
1–2	105 (53.9)	102 (55.1)
≥ 3	57 (29.2)	44 (23.8)
Age at first birth^*c*^ (years)	26.5 ± 4.4	26.7 ± 5.4	0.81
Menopausal status			0.43
Premenopausal	28 (14.3)	32 (17.2)
Postmenopausal	168 (85.7)	154 (82.8)
Use of hormone replacement therapy			0.46
Never	177 (94.7)	167 (92.8)
Ever	10 (5.3)	13 (7.2)
Previous breast biopsy			0.02
No	189 (96.4)	168 (90.8)
Yes	7 (3.6)	17 (9.2)
Family history of breast cancer			0.27
No	166 (84.6)	147 (79.0)
Second-degree relative	15 (7.7)	16 (8.6)
First-degree relative	15 (7.7)	23 (12.4)
Serum total lipids (mg/mL)	7.67 ± 1.87	7.42 ± 1.60	0.17
Serum TEXB-α^*d*^ (Eeq pM/mL)	2.99 (7.86)	8.32 (5.72)	< 0.001
Undetermined estrogenicity	90 (45.9)	79 (42.5)	0.50
Serum TEXB-β^*d*^ (Eeq pM/mL)	5.96 (5.65)	9.94 (4.57)	0.01
Undetermined estrogenicity	70 (35.7)	64 (34.4)	0.79
Serum PCB-138^*e*^ (ng/mL)	0.89 (3.25)	1.04 (3.13)	0.21
Serum PCB-153^*e*^ (ng/mL)	1.37 (3.28)	1.62 (2.92)	0.15
Serum PCB-180^*e*^ (ng/mL)	0.72 (3.07)	0.71 (2.71)	0.97
Serum HCB^*e*^ (ng/mL)	0.68 (3.40)	0.53 (4.11)	0.06
Serum *p*,*p’*-DDE^*e*^ (ng/mL)	2.69 (5.04)	2.45 (4.42)	0.56
Abbreviations: Eeq pM/mL, estradiol equivalent in picomolar per milliliter of serum; HCB, hexachlorobenzene; *p*,*p’*-DDE, *p*,*p’*-dichlorodiphenyldichloroethylene; PCB, polychlorinated biphenyl; TEXB, total effective xenoestrogen burden. Values are the mean ± SD or number (percentage). ^***a***^*p*-Value for homogeneity of means or proportions between breast cancer cases and controls. ^***b***^Body mass index and smoking status 1 year before baseline interview. ^***c***^Age at first birth among parous women. ^***d***^Geometric mean (geometric SD) serum levels of the total effective xenoestrogen burden of α (TEXB-α) and β (TEXB-β) fractions, together with numbers (percentages) of samples with undetermined estrogenicity in the bioassay. ^***e***^Geometric mean (geometric SD) serum concentrations of PCB-138, PCB-153, PCB-180, HCB, and *p*,*p’*-DDE.			

Serum levels of TEXB-α and TEXB-β were moderately correlated among controls (Pearson correlation coefficient for log-transformed variables: 0.34; 95% CI: 0.14, 0.51). Serum concentrations of PCB-138, PCB-153, PCB-180, HCB, and *p*,*p*´-DDE were weakly correlated with TEXB-α levels among controls (Pearson correlations for log-transformed variables of –0.21, –0.01, –0.18, –0.18, and –0.17, respectively) and were virtually uncorrelated with TEXB-β levels (–0.05, –0.03, –0.07, –0.02, and 0.03). Apart from differences by geographic region, no other significant trend in breast cancer risk factors or serum chemical concentrations was observed across tertiles of serum TEXB-α and TEXB-β levels among controls, in part because of the limited number of control women within each tertile (Table 2). Compared with controls with determined estrogenicity in serum samples, controls with undetermined TEXB-α and TEXB-β had similar risk factor distributions but had significantly higher serum concentrations of PCB-138, PCB-180, HCB, and *p*,*p*´-DDE (Table 2).

**Table 2:** Main characteristics and serum concentrations of specific organohalogenated compounds by tertile of total effective xenoestrogen burden of alpha (TEXB-α) and beta (TEXB-β) fractions among controls (*n* = 196).

Characteristic	Serum TEXB-α^*a*^ (Eeq pM/mL)						Serum TEXB-β^*a*^ (Eeq pM/mL)					
	Tertile 1 (≤ 2.62)	Tertile 2 (2.63–8.75)	Tertile 3 (≥ 8.76)	*p* for trend^*b*^	Undetermined estrogenicity	*p*-Value^*c*^	Tertile 1 (≤ 4.56)	Tertile 2 (4.57–11.27)	Tertile 3 (≥ 11.28)	*p* for trend^*b*^	Undetermined estrogenicity	*p*-Value^*c*^
No. of control women	35	36	35		90		42	42	42		70
Median serum level (Eeq pM/mL)	0.07	4.78	15.18				2.29	7.41	19.89
Province				0.002		< 0.001				0.08		< 0.001
Madrid	31.4	61.1	48.6		37.8		47.6	50.0	61.9		24.3
Barcelona	17.2	2.8	14.3		24.4		11.9	14.3	2.4		31.4
Navarra	11.4	27.8	25.7		3.3		19.1	11.9	28.6		1.4
Cantabria	40.0	8.3	11.4		34.5		21.4	23.8	7.1		42.9
Age (years)	57.0	61.8	58.9	0.73	60.5	0.40	57.7	60.6	59.9	0.46	60.7	0.40
Body mass index (kg/m^2^)	27.5	25.3	26.5	0.65	25.9	0.39	26.0	25.5	26.5	0.50	26.4	0.54
High school education or more	62.9	44.4	54.3	0.68	50.0	0.60	57.1	52.4	59.5	0.72	44.3	0.11
Ever smoker	45.7	36.1	37.1	0.55	33.3	0.36	35.7	40.5	38.1	0.90	34.3	0.60
Nulliparous	22.9	11.1	14.3	0.45	18.0	0.72	14.3	19.0	7.1	0.21	23.2	0.09
Age at first birth (years)	26.1	26.9	26.8	0.70	26.4	0.74	26.8	27.0	27.0	0.88	25.7	0.08
Postmenopausal	71.4	94.4	88.6	0.11	86.7	0.73	81.0	85.7	92.9	0.10	84.3	0.67
Ever use of hormone therapy	3.1	3.0	2.9	0.95	8.0	0.12	7.1	2.4	7.1	0.80	4.8	0.83
Previous breast biopsy	2.9	5.6	0.0	0.30	4.4	0.54	7.1	4.8	0.0	0.05	2.9	0.68
Family history of breast cancer	25.7	16.7	17.1	0.45	10.0	0.05	16.7	19.0	21.4	0.59	8.6	0.04
Serum total lipids (mg/mL)	7.73	7.36	7.93	0.51	7.66	0.97	7.35	8.04	7.82	0.47	7.54	0.50
Serum PCB-138^*d*^ (ng/mL)	0.98	0.77	0.58	0.11	1.09	0.03	0.73	0.95	0.66	0.55	1.17	0.02
Serum PCB-153^*d*^ (ng/mL)	1.16	1.51	1.27	0.91	1.45	0.56	1.18	1.88	1.11	0.53	1.40	0.83
Serum PCB-180^*d*^ (ng/mL)	0.61	0.42	0.60	0.82	1.01	< 0.001	0.56	0.69	0.53	0.67	1.03	0.001
Serum HCB^*d*^ (ng/mL)	0.62	0.56	0.45	0.30	0.90	0.003	0.53	0.83	0.42	0.19	0.93	0.007
Serum *p*,*p*´-DDE^*d*^ (ng/mL)	2.69	1.37	1.54	0.26	4.37	< 0.001	1.45	3.08	1.89	0.82	4.42	0.001
Abbreviations: Eeq pM/mL, estradiol equivalent in picomolar per milliliter of serum; HCB, hexachlorobenzene; *p*,*p’*-DDE, *p*,*p’*-dichlorodiphenyldichloroethylene; PCB, polychlorinated biphenyl; TEXB-α, total effective xenoestrogen burden of α fraction; TEXB-β, total effective xenoestrogen burden of beta fraction. Values are means or percentages. ^***a***^Tertiles of TEXB-α and TEXB-β, together with serum samples with undetermined estrogenicity in the bioassay. ^***b***^*p*-Value for linear trend in means or proportions across tertiles of the total effective xenoestrogen burden of α and β fractions except for province, which corresponds to the *p*-value for homogeneity of province distributions among tertiles. ^***c***^*p*-Value for homogeneity of means or proportions comparing serum samples with determined estrogenicity in the bioassay (all tertiles combined) with samples with undetermined estrogenicity. ^***d***^Geometric mean serum concentrations of PCB-138, PCB-153, PCB-180, HCB, and *p*,*p*´-DDE.												

**Figure 1:**
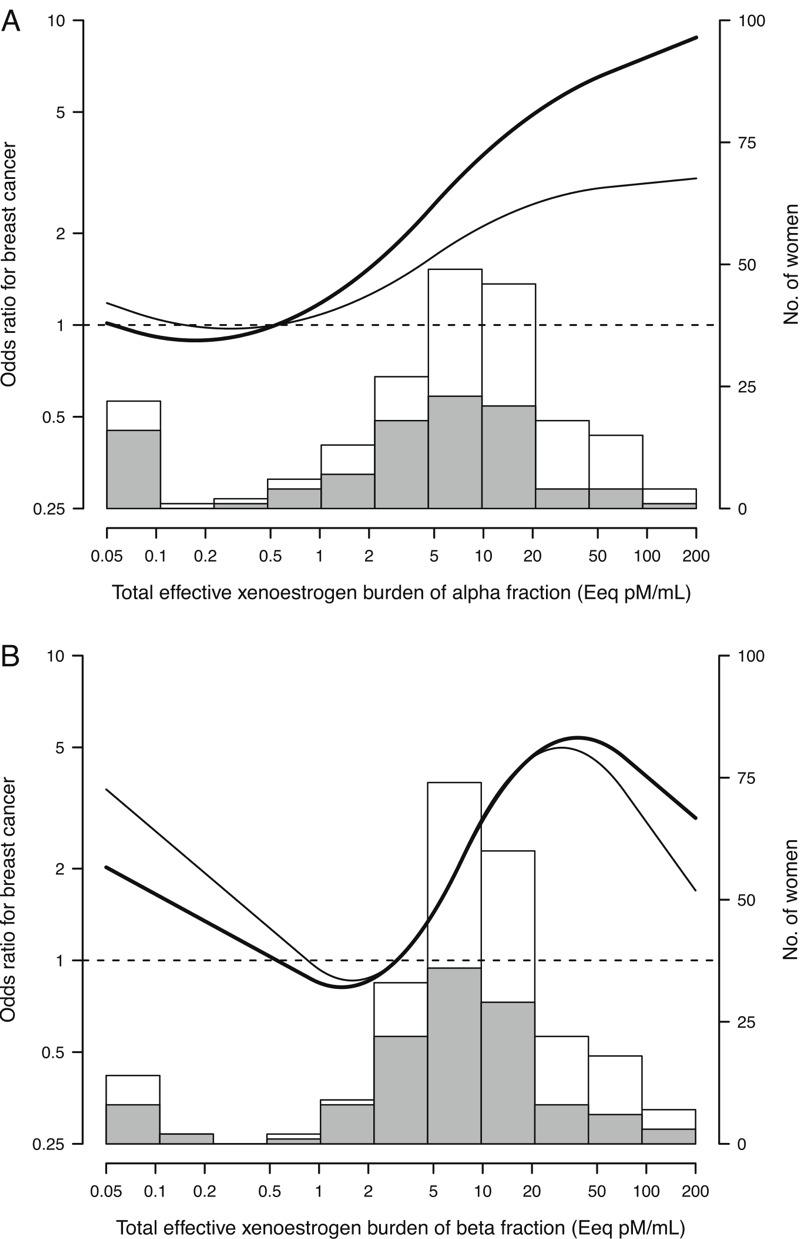
Odds ratios for breast cancer by serum levels of total effective xenoestrogen burden of alpha (*A*) and beta (*B*) fractions. Curves represent adjusted odds ratios based on restricted quadratic splines for log-transformed levels of total effective xenoestrogen burden of alpha and beta fractions with knots at the 10th, 50th, and 95th percentiles. The reference value (odds ratio = 1) was set at the 20th percentile of each fraction distribution among controls (0.54 and 2.97 Eeq pM/mL for alpha and beta fractions, respectively). Odd ratios were adjusted for province, age, body mass index, education level, serum total lipid levels, smoking status, number of births, age at first birth, menopausal status, use of hormone replacement therapy, previous breast biopsy, and family history of breast cancer (bold curves), and further adjusted for the other fraction of total effective xenoestrogen burden (thin curves). Histograms represent each fraction distribution among controls (shaded bars) and breast cancer cases (white bars).

In models adjusted for sociodemographic and traditional breast cancer risk factors (Table 3), the risk for breast cancer increased with increasing serum levels of both TEXB-α and TEXB-β (*p* for linear trend = 0.003 and 0.001, respectively). Compared with the first tertile, the odds ratios for the second and third tertiles of serum TEXB-α were 1.77 (95% CI: 0.76, 4.10) and 3.45 (95% CI: 1.50, 7.97), and those for the second and third tertiles of serum TEXB-β were 2.35 (95% CI: 1.10, 5.03) and 4.01 (95% CI: 1.88, 8.56). The increase in breast cancer risk was marked and sustained over all serum TEXB-α levels > 0.5 Eeq pM/mL (Figure 1A). However, a sigmoidal risk trend was observed across serum TEXB-β levels, with a sharp increase in risk between 2 and 40 Eeq pM/mL and a downturn at higher levels (Figure 1B). When serum TEXB-α and TEXB-β levels were mutually adjusted for each other, the association of TEXB-α with breast cancer risk was substantially attenuated, whereas that for TEXB-β remained virtually unchanged (Table 3 and Figure 1). The risk for breast cancer did not differ among women with undetermined versus determined estrogenicity (fully adjusted odds ratios of 0.73 and 0.97, respectively) (Table 3).

**Table 3:** Odds ratios for breast cancer (95% confidence intervals) by tertile of total effective xenoestrogen burden of alpha and beta fractions (*n* = 382).

TEXB	Tertile 1	Tertile 2	Tertile 3	*p* for trend^*a*^	Undetermined estrogenicity^*b*^
Serum TEXB-α^*c*^ (Eeq pM/mL)	≤ 2.62	2.63–8.75	≥ 8.76
Number of controls/breast cancer cases	35/18	36/32	35/57		90/79
Model 1^*d*^	1.00 (Reference)	1.64 (0.74, 3.62)	3.04 (1.38, 6.70)	0.005	0.83 (0.52, 1.32)
Model 2^*e*^	1.00 (Reference)	1.77 (0.76, 4.10)	3.45 (1.50, 7.97)	0.003	0.73 (0.45, 1.20)
Model 3^*f*^	1.00 (Reference)	1.50 (0.55, 4.08)	1.80 (0.63, 5.09)	0.32
Serum TEXB-β^*c*^ (Eeq pM/mL)	≤ 4.56	4.57–11.27	≥ 11.28
Number of controls/breast cancer cases	42/21	42/43	42/58		70/64
Model 1^*d*^	1.00 (Reference)	2.14 (1.06, 4.35)	3.27 (1.62, 6.61)	0.002	0.86 (0.52, 1.41)
Model 2^*e*^	1.00 (Reference)	2.35 (1.10, 5.03)	4.01 (1.88, 8.56)	0.001	0.97 (0.58, 1.65)
Model 3^*f*^	1.00 (Reference)	1.75 (0.65, 4.71)	3.53 (1.24, 10.0)	0.02
Abbreviations: Eeq pM/mL, estradiol equivalent in picomolar per milliliter of serum; TEXB-α, total effective xenoestrogen burden of α fraction; TEXB-β, total effective xenoestrogen burden of beta fraction. ^***a***^*p*-Value for linear risk trend across tertiles based on an ordinal variable with the median level of each tertile. ^***b***^Odds ratio for breast cancer comparing women with undetermined estrogenicity in the bioassay with all other women with determined estrogenicity. ^***c***^Serum levels of TEXB-α and TEXB-β. ^***d***^Adjusted for province (Madrid, Barcelona, Navarra, or Cantabria), age (continuous), body mass index (continuous), education level (primary or less, high school, or college), and serum total lipid levels (continuous). ^***e***^Further adjusted for smoking status (never, former, or current), number of births (nulliparous, 1–2, or ≥ 3), age at first birth (continuous), menopausal status (premenopausal or postmenopausal), use of hormone replacement therapy (never or ever), previous breast biopsy (no or yes), and family history of breast cancer (no, second-degree relative, or first-degree relative). ^***f***^Further adjusted for the other fraction of total effective xenoestrogen burden (tertiles).					

Individual organohalogenated xenoestrogens contained in the α fraction showed weak and opposing associations with breast cancer risk (Table 4). In models adjusted for sociodemographic and traditional risk factors, the odds ratios for breast cancer comparing the third with the first tertile were 1.73 (95% CI: 0.96, 3.14) for PCB-138, 1.36 (95% CI: 0.75, 2.45) for PCB-153, 1.01 (95% CI: 0.55, 1.87) for PCB-180, 0.84 (95% CI: 0.45, 1.58) for *p*,*p*´-DDE, and 0.60 (95% CI: 0.32, 1.15) for HCB.

**Table 4:** Odds ratios for breast cancer (95% confidence intervals) by tertile of specific organohalogenated compounds (*n* = 382).

Organohalogenated compound	Tertile 1	Tertile 2	Tertile 3	*p* for trend^*a*^
Serum PCB-138^*b*^ (ng/mL)	≤ 0.80	0.81–1.59	≥ 1.60
No. of controls/breast cancer cases	65/52	65/60	66/74
Model 1^*c*^	1.00 (Reference)	1.27 (0.75, 2.14)	1.63 (0.93, 2.85)	0.09
Model 2^*d*^	1.00 (Reference)	1.30 (0.74, 2.27)	1.73 (0.96, 3.14)	0.07
Model 3^*e*^	1.00 (Reference)	1.34 (0.64, 2.81)	1.64 (0.78, 3.46)	0.20
Serum PCB-153^*b*^ (ng/mL)	≤ 0.90	0.91–2.07	≥ 2.08
No. of controls/breast cancer cases	63/50	68/78	65/58
Model 1^*c*^	1.00 (Reference)	1.54 (0.92, 2.58)	1.21 (0.69, 2.12)	0.85
Model 2^*d*^	1.00 (Reference)	1.42 (0.83, 2.42)	1.36 (0.75, 2.45)	0.46
Model 3^*e*^	1.00 (Reference)	0.90 (0.45, 1.82)	1.33 (0.64, 2.75)	0.36
Serum PCB-180^*b*^ (ng/mL)	≤ 0.52	0.53–1.17	≥ 1.18
No. of controls/breast cancer cases	65/63	66/56	65/67
Model 1^*c*^	1.00 (Reference)	0.82 (0.48, 1.41)	1.04 (0.59, 1.85)	0.73
Model 2^*d*^	1.00 (Reference)	0.82 (0.46, 1.43)	1.01 (0.55, 1.87)	0.81
Model 3^*e*^	1.00 (Reference)	0.96 (0.47, 1.98)	1.09 (0.49, 2.43)	0.81
Serum HCB^*b*^ (ng/mL)	≤ 0.43	0.44–1.25	≥ 1.26
No. of controls/breast cancer cases	65/75	66/58	65/53
Model 1^*c*^	1.00 (Reference)	0.69 (0.41, 1.15)	0.56 (0.30, 1.02)	0.09
Model 2^*d*^	1.00 (Reference)	0.69 (0.41, 1.18)	0.60 (0.32, 1.15)	0.18
Model 3^*e*^	1.00 (Reference)	0.63 (0.32, 1.24)	0.64 (0.27, 1.50)	0.38
Serum *p*,*p’*-DDE^*b*^ (ng/mL)	≤ 1.37	1.38–6.76	≥ 6.77
No. of controls/breast cancer cases	65/56	66/86	65/44
Model 1^*c*^	1.00 (Reference)	1.50 (0.90, 2.49)	0.72 (0.40, 1.31)	0.06
Model 2^*d*^	1.00 (Reference)	1.59 (0.94, 2.70)	0.84 (0.45, 1.58)	0.20
Model 3^*e*^	1.00 (Reference)	1.61 (0.81, 3.21)	0.63 (0.27, 1.46)	0.10
HCB, hexachlorobenzene; *p*,*p’*-DDE, *p*,*p’*-dichlorodiphenyldichloroethylene; PCB, polychlorinated biphenyl. ^***a***^*p*-Value for linear risk trend across tertiles based on an ordinal variable with the median level of each tertile. ^***b***^Serum concentrations of PCB-138, PCB-153, PCB-180, HCB, and *p*,*p’*-DDE. ^***c***^Adjusted for province (Madrid, Barcelona, Navarra, or Cantabria), age (continuous), body mass index (continuous), education level (primary or less, high school, or college), and serum total lipid levels (continuous). ^***d***^Further adjusted for smoking status (never, former, or current), number of births (nulliparous, 1–2, or ≥ 3), age at first birth (continuous), menopausal status (premenopausal or postmenopausal), use of hormone replacement therapy (never or ever), previous breast biopsy (no or yes), and family history of breast cancer (no, second-degree relative, or first-degree relative). ^***e***^Further adjusted for the total effective xenoestrogen burden of beta fraction (tertiles).				

**Figure 2:**
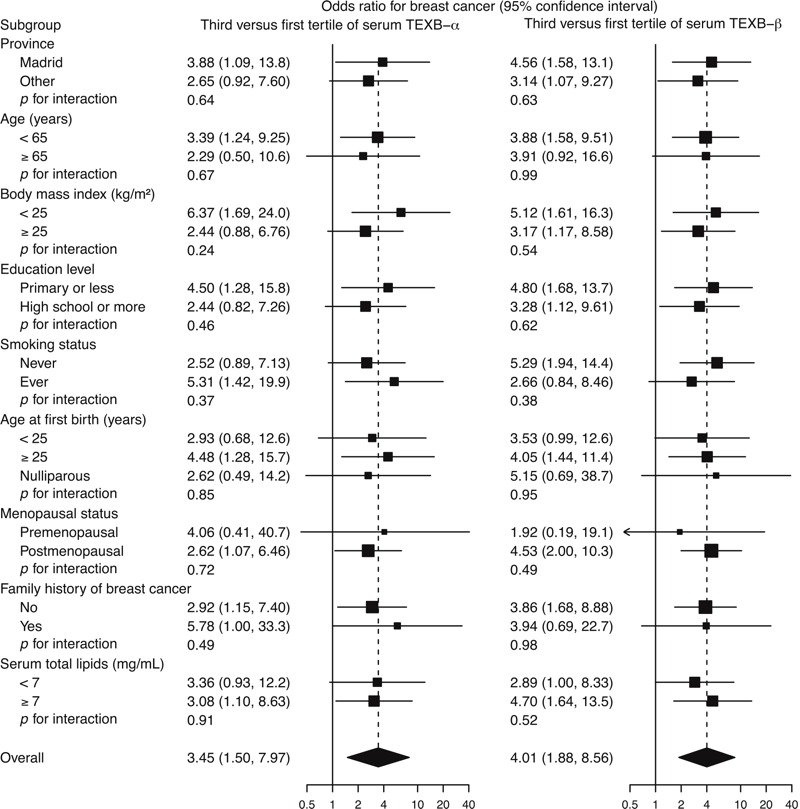
Odds ratios for breast cancer comparing the third with the first tertile of total effective xenoestrogen burden of α (TEXB-α) and β (TEXB-β) fractions by subgroup. Subgroup-specific odds ratios (squares with area inversely proportional to the variance) and their 95% confidence intervals (horizontal lines) were obtained from logistic regression models with interaction terms of serum TEXB-α and TEXB-β tertiles with the corresponding subgroup indicators and were adjusted for province, age, body mass index, education level, serum total lipid levels, smoking status, number of births, age at first birth, menopausal status, use of hormone replacement therapy, previous breast biopsy, and family history of breast cancer.

In subgroup analyses, the increased risk for breast cancer in the third versus the first tertile of serum TEXB-α tended to be higher in women with normal weight and those with a family history of breast cancer (subgroup-specific odds ratios of 6.37 and 5.78, respectively), although none of these effect modifications was statistically significant (Figure 2). The positive association of serum TEXB-β with breast cancer risk was quite homogeneous across all subgroups.

## Discussion

This is the first study to show a graded positive association between serum total xenoestrogen burden, as determined by the α fraction of the TEXB bioassay, and breast cancer risk. Women in the third tertile of serum TEXB-α had a 3.45-fold increase in breast cancer risk compared with those in the first tertile. The β fraction of the TEXB bioassay was also positively associated with the risk for breast cancer; this result was somewhat expected given that circulating endogenous estrogens were included in this fraction. Finally, none of the individual organohalogenated xenoestrogens analyzed in this study was significantly associated with breast cancer risk.

Most studies in this field have focused on serum or adipose concentrations of a single chemical or a small number of chemicals, ignoring the cumulative effects of mixtures. Recognizing this limitation, the World Health Organization Report has concluded that it is critical to move beyond the analysis of one chemical at a time to explore the effects of EDC mixtures (WHO/UNEP 2013). The TEXB bioassay is an alternative technique that directly measures the combined estrogenic effect of all compounds included in either α or β HPLC fractions. Because additive, synergistic, or antagonistic mechanisms may be present in these complex mixtures (Evans et al. 2012; Scholze et al. 2014), this approach constitutes a more efficient way to explore the cumulative impact of these compounds. In fact, cell culture studies have shown that EDC mixtures can produce a significant proliferative effect even at concentrations of individual chemicals that alone do not produce detectable effects (Rajapakse et al. 2002). Thus, the estrogenic potential of EDCs, when tested individually, is likely to be underestimated (Kortenkamp 2007).

Most women in this study (83.5%) had detectable serum concentrations of all of the measured organochlorine chemicals, reflecting the ubiquity of their exposure in the general population. We found no differences in PCB, HCB, or *p*,*p*´-DDE concentrations between cases and controls, and none of these single compounds was positively correlated with serum TEXB-α levels among controls, reflecting their modest contribution to the total xenoestrogen burden. Similarly, a previous study reported no correlation between individual organohalogenated xenoestrogens and their combined estrogenic activity in adipose tissue (Ibarluzea et al. 2004).

**Figure 3:**
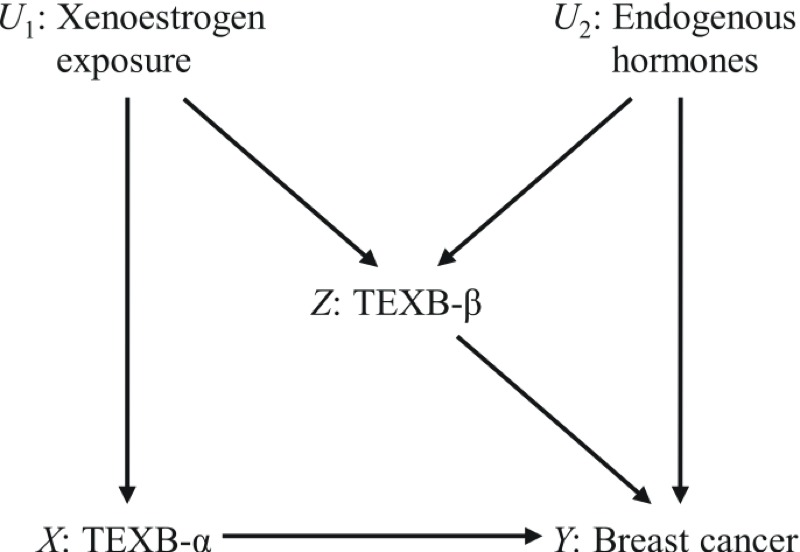
Diagram with causal relationships among total effective xenoestrogen burden of α (TEXB-α) and β (TEXB-β) fractions, breast cancer, and unmeasured exposures to xenoestrogens and endogenous hormones. The causal path from TEXB-α to breast cancer *X*→*Y* is confounded by the indirect path *X*←*U*_1_→*Z*→*Y*, which can be blocked by adjusting for TEXB-β. However, conditioning on TEXB-β unblocks the other indirect path *X*←*U*_1_→*Z*←*U*_2_→*Y*, thus resulting in selection bias.

The extensive HPLC fractionation performed before the TEXB bioassay was designed to separate organohalogenated lipophilic xenoestrogens in the α fraction from endogenous hormones and from more polar xenoestrogens in the β fraction (Fernández et al. 2004). In our study, serum TEXB-α and TEXB-β levels were positively correlated among controls, which resulted in an attenuation of the association between TEXB-α and breast cancer risk after adjusting for TEXB-β. The causal diagram in Figure 3 displays the assumed causal relationships among TEXB-α, TEXB-β, breast cancer, and other relevant exposures, providing a valuable tool for identifying potential sources of bias and their control. Serum TEXB-α and TEXB-β levels are assumed to be affected by an unspecified common exposure to both lipophilic and polar xenoestrogens (Fernández et al. 2004), as suggested by the observed association between TEXB-α and TEXB-β. Serum TEXB-β levels were also affected by unmeasured endogenous hormones, which are independent of xenoestrogen exposure and directly influence breast cancer risk. According to this diagram, the causal effect of TEXB-α on breast cancer is confounded by correlated xenoestrogens present in the β fraction, whose upward bias can be controlled by adjusting for TEXB-β. However, this adjustment induces a negative conditional association between xenoestrogens and endogenous hormones, which results in a downward selection bias that can be as severe as the controlled confounding if endogenous hormone effects are strong (Greenland 2003). Thus, without further information on xenoestrogen exposure or endogenous hormones, we can only conclude that the underlying effect of TEXB-α on breast cancer lies between the estimated associations with and without adjustment for TEXB-β.

Although the risk for breast cancer increased progressively across all detected TEXB-α levels > 0.5 Eeq pM/mL, the association for TEXB-β followed a sigmoidal trend, with a sharp increase in risk between 2 and 40 Eeq pM/mL and a downturn at higher levels. Nonmonotonic responses are remarkably common in studies of natural hormones and EDCs (Engström et al. 2015; Vandenberg et al. 2012). The upward-then-downward risk trend for TEXB-β could be explained by receptor competition between endogenous hormones and polar xenoestrogens included in the β fraction (Vandenberg et al. 2012). At low to intermediate TEXB-β levels, natural hormone concentrations do not saturate receptors, and xenoestrogens bind to unoccupied receptors to increase the overall cellular response; however, at high TEXB-β levels, xenoestrogens can outcompete natural ligands and, because of their weaker estrogenic activity, result in an attenuation of the overall biological response.

Although the present study had limited power to detect effect modifications, we observed somewhat greater effects of TEXB-α on breast cancer risk in women of normal weight and in those with a family history of breast cancer. A stronger effect in leaner women was also evident in a previous case–control study using the TEXB bioassay in adipose tissue (Ibarluzea et al. 2004); in that study, the effect was attributed to a greater relative impact of EDCs in women with lower levels of endogenous hormones accumulated in their fat. With regard to the stronger association between TEXB-α and breast cancer risk in women with a family history of breast cancer, this group was too small to draw further conclusions, and larger studies are needed to confirm this potential effect–measure modification.

Contrary to previous findings in adipose tissue extracts (Fernández et al. 2004; Ibarluzea et al. 2004), the combined estrogenic activity in serum samples was not associated with age, body mass index, or any other characteristic in our study, with the exception of the differences observed by geographical region. Control women from the province of Navarra had higher than the overall average TEXB-α and TEXB-β levels, whereas those from Cantabria presented lower than the overall average estrogenic activity in both fractions. We have no clear explanation for these geographical differences. Previous studies have reported higher than the overall average serum concentrations of PCBs in healthy adults from the northern Spanish regions (Agudo et al. 2009; Huetos et al. 2014) and elevated HCB levels in the province of Navarra (Jakszyn et al. 2009). However, these single chemicals contributed little to the total xenoestrogen burden in the women in this study and can hardly explain the observed regional variations. Navarra is also one of the regions in Spain with a higher than average prevalence of postmenopausal hormone therapy use (Isidoro et al. 2015), but our study found no differences in TEXB-α or TEXB-β levels between never and ever users of hormone therapy. Thus, larger population-based studies are required to identify determinants of serum TEXB levels that contribute to their geographical distribution.

The strengths of this study include the population-based case–control design and the use of a reliable biomarker for the combined estrogenic effect of EDC mixtures. However, several limitations must be mentioned. First, the response rate among population controls was moderate, with higher participation rates among women with higher levels of education. To control for this potential selection bias, all analyses were adjusted for education level. Second, owing to the case–control design, serum samples were collected after diagnosis in breast cancer cases, which might have led to a reverse causation bias if serum concentrations of hormones or xenoestrogens had changed after disease onset. To minimize the potential for reverse causation, we restricted the analysis to patients with incident breast cancer who did not receive neoadjuvant chemotherapy or hormone therapy before blood extraction. However, because most growing breast tumors are estrogen-demanding, serum estrogen levels might have decreased after disease onset, leading to a potential dilution in the associations, particularly for TEXB-β because endogenous hormones have higher binding affinity to estrogen receptors and shorter biological half-lives than xenoestrogens. Third, adipose tissue extracts were not collected in our study, and TEXB in serum samples was taken as a surrogate of the overall estrogenic activity at the mammary gland. Although many EDCs are lipophilic and accumulate in breast fatty tissue, their concentrations in serum are relatively low and depend on serum lipid content. For this reason, all analyses relating serum TEXB levels to breast cancer risk were adjusted for serum total lipids. Fourth, estrogenicity could not be determined in over one third of the serum samples because breast cancer cells treated with their extracts grew less than did steroid-free control cells in the TEXB bioassay. Although there is no clear explanation for this lack of growth, samples with undetermined estrogenicity had significantly higher levels of all measured organohalogenated xenoestrogens; therefore, they might also have had elevated concentrations of other unmeasured common-source xenobiotics that prevented or hampered cellular growth. Nevertheless, because undetermined estrogenicity was unrelated to case–control status, our analyses based on determined samples will provide an unbiased estimate of the association between TEXB and breast cancer risk. Finally, the inherent time-consuming and serum-demanding characteristics of the TEXB bioassay, together with the substantial proportion of undetermined samples, greatly limited the effective sample size and power of the present study, which precluded more extensive analyses according to tumor subtypes.

## Conclusions

The combined estrogenic activity of mixtures of organohalogenated xenoestrogens in serum samples was positively associated with breast cancer risk, even though no single compound showed a significant effect when analyzed individually. The increase in risk was strong and progressive across all detected estrogenic levels. Our findings show the importance of evaluating mixtures of EDCs, rather than single chemicals, in epidemiological studies on hormone-related cancers. This study provides new evidence linking breast cancer to combined exposures to EDCs, something to be considered by policy agencies in charge of controlling their production and distribution.
